# Food beliefs and practices among the Kalenjin pregnant women in rural Uasin Gishu County, Kenya

**DOI:** 10.1186/s13002-017-0157-8

**Published:** 2017-05-25

**Authors:** Roselyter Monchari Riang’a, Jacqueline Broerse, Anne Kisaka Nangulu

**Affiliations:** 1 Moi University, School of Arts and Social Sciences, P.O. Box 3900-30100, Eldoret, Kenya; 2 Athena Institute, Faculty of Earth and Life Sciences, VU University Amsterdam, De Boelelaan 1085, 1081 HV Amsterdam, The Netherlands; 3 Commission for University Education, Red Hill Road, off Limuru Road, Gigiri, P.O. Box 54999 – 00200, Nairobi Kenya

**Keywords:** Food beliefs, Taboos, Maternal and child health, Nutritional interventions, Pregnancy complications, Kalenjin, Kenya

## Abstract

**Background:**

Understanding food beliefs and practices is critical to the development of dietary recommendations, nutritional programmes, and educational messages. This study aimed to understand the pregnancy food beliefs and practices and the underlying reasons for these among the contemporary rural Kalenjin communities of Uasin Gishu County, Kenya.

**Methods:**

Through semi-structured interviews, data was collected from 154 pregnant and post-natal Kalenjin women about restricted and recommended foods, and why they are restricted or recommended during pregnancy. Respondents were purposively selected (based on diversity) from those attending Maternal and Child Health (MCH) care in 23 rural public health facilities. Key informant interviews *(n* = 9) with traditional Birth Attendants (TBA) who were also herbalists, community health workers, and nursing officers in charge of MCH were also conducted. Quantitative data was analysed using SPSS software. Data from respondents who gave consent to be tape recorded (*n* = 42) was transcribed and qualitatively analysed using MAXQDA software.

**Results:**

The restriction of animal organs specifically the tongue, heart, udder and male reproductive organs, meat and eggs, and the recommendation of traditional green vegetables and milk was reported by more than 60% of the respondents. Recommendation of fruits, traditional herbs, *ugali* (a dish made of maize flour, millet flour, or Sorghum flour, sometimes mixed with cassava flour), porridge and liver, and restriction of avocadoes and oily food were reported by more than 20% of the respondents. The reasons for observing these dietary precautions were mainly fears of: big foetuses, less blood, lack of strength during birth, miscarriages or stillbirths, and maternal deaths as well as child’s colic and poor skin conditions after birth.

**Conclusion:**

Pregnancy food beliefs were widely known and practised mainly to protect the health of the mother and child, and ensuring successful pregnancy outcome. Given the deep-rooted nature of the beliefs, it is advisable that when nutritious foods are restricted, nutritional interventions should rather search for alternative sources of nutrition which are available and considered to be appropriate for pregnancy. On the other hand, nutritional advice that does not address these health concerns and assumptions that underlie successful pregnancy and delivery is unlikely to be effective.

## Background

Undernutrition is a serious global health problem which has not yet been sufficiently addressed. Approximately 795 million people are undernourished, mostly Low and Middle-Income Countries (LMICs) [1]. Nutrition is most critical during pregnancy because poor nutrition puts both mother and baby at risk. Poorly nourished expectant mothers are at a higher risk of having a preterm birth, and giving birth to babies with low birth-weight and Intra Uterine Growth Restrictions (IUGR); while also facing multiple threats to their own health and survival [2, 3]. Babies born underweight, pre-term or with IUGR have increased risk of neonatal death. A number of those who survive these conditions may develop into stunted adults who will, in turn, give birth to stunted children, representing a vicious cycle of nutritional problems [4]. Nearly half (45%) of global deaths among children under five are attributed to undernutrition, amounting to 3 million deaths each year [5]. In Kenya, 35% of children under five have been found to be stunted with higher levels of stunting in rural areas [6]. Furthermore, 56% of infant deaths in Kenya occur during the first month of life and 5% of children do not survive to the age of 5 years [7].

In LMICs, socio-economic factors (especially consumer income) have been associated with inadequate nutritional status and household food security [8–10]. However, a study undertaken in Ethiopia established that some of the highest prevalence rates of malnutrition were found in the food-surplus regions of the country, indicating that food availability is only one component of food security and that it does not necessarily ensure adequate nutritional status [11]. Similarly in Kenya, although 89% of land is arid and semi-arid [12], even the arable regions still experience nutritional deficiencies. Uasin Gishu County for instance, where this study was conducted, has climatic conditions and soil type which are generally suitable for livestock keeping and food crop production with an average rural land holding of 5 hectares, hence it is commonly known as the country’s food basket [13]. However, the nutritional status of Uasin Gishu County is worse than the national norm. Some 11.5% of the Uasin Gishu population is underweight compared to the national level (11.0%) while 31.2% of children are stunted compared to 26.0% nationally [7].

The UN Children’s Fund (UNICEF) Food-Care Health conceptual framework lists cultural norms, taboos and beliefs as one factor that may cause malnutrition [14]. Meyer-Rochow [15] for instance, established that food items within a given ecological zone may be considered inedible due to “nutritional taboos.” Food beliefs and taboos are a global phenomenon that are intended to have a positive effect to the practising community including: conservation of a scarce or sacred resource [15, 16], maintenance of social norms, morals, group cohesion and identity [17] and protection of human health [18, 19]. Food taboos and restrictions are particularly strictly observed during pregnancy as pregnant women are considered to be more vulnerable and hence what they eat must be regulated to protect the foetus and reduce the occurrence of complicated labour and delivery [19–25]. Other studies, however, indicate that failure to make use of available food resources in a given ecological zone due to taboos and beliefs is one of the major causes of malnutrition [26, 27]. This indicates that more research needs to be carried out on this crucial area.

In Kenya, only a few studies have concentrated on the nexus between pregnancy, culture and nutrition. Boerma and Mati [28] and Oniango and Komokoti [29] are among the few that have researched this subject. They established that pregnant women are restricted from eating certain food items such as liver, intestines, kidney, milk, sweet potatoes, sugar, salt, eggs and bananas because these foods are believed to cause obstructed labour. The same norms, however, encourage pregnant women to take certain food items such as cow’s blood, sour milk and lots of water, and to vomit following a heavy meal. These norms are designed to keep the baby small at birth and thus ensure safe delivery for both mother and baby.

This clearly indicates the existence of food beliefs related to pregnancy in Kenya. There are 42 ethnic groups in Kenya with varying and changing nutritional traditions and taboos which indicates the need to develop an elaborate body of knowledge concerning diverse cultural dietary practices during pregnancy as a basis for contextualised understanding and effective interventions. This is a particularly important issue in areas with high levels of child stunting, such as the area under study. Lack of appropriate knowledge on culturally prescribed nutritional taboos and beliefs can have a powerful impact on the outcome of malnutrition relief efforts or prevention campaigns and interventions [20]. This study investigates food belief practices among the contemporary Kalenjin community of rural Uasin Gishu County. The specific objectives were to investigate the various food beliefs during pregnancy, and examine the underlying reasons. The findings of this study will provide a basis for developing culturally appropriate nutritional interventions and empowerment programmes with a view to providing effective nutritional counselling targeting pregnant women. This may improve birth outcomes and long-term quality of life.

## Methods

### Theoretical framework (symbolic interactionist theory (SIT))

The research findings of this study were interpreted and understood through the symbolic interactionist perspective as expounded by Messer [30]. Symbolic interaction theory (SIT) is a frame of reference for how people act toward things based on the meaning those things have for them, and these meanings are derived from social interaction and modified through interpretation [31]. Messer (1989) classified food according to a number of symbolic dimensions which are culturally constructed in the process of social interaction and this influences human food selection. Having no biological base, these social constructs of nutrition are based on what people believe to be true about food and not just on what is objectively true. So, the symbolic meaning of food overrides those actual biological facts regarding diet and health. These may be the dimensions most prominent in the food proscriptions and prescriptions of nutritionally "vulnerable groups" such as infants, children, and pregnant and lactating women. Messer [30] found that while people shared the same general structure and rules for classification, they did not necessarily judge all items equivalently, given the differences in individual experience. Also, individuals differed in the extent to which they had acquired information and applied it to their own diets and health. Understanding these food symbolic dimensions therefore can greatly aid in interpretation of food habits that might be beneficial or harmful to a particular population or subpopulation.

Messer [30] classified food into eight cultural symbolic dimensions including: hot-cold, health, age, gender, illness, rituals and economic status. These symbolic classifications influence food selection, food preferences, and dietary intakes. In this study, we adopted these symbolic classifications as guiding principles in the analysis of the research findings. However, hot-cold and economic status factors were excluded because they were not established in this study. On the other hand, symbolic classfication of food as "traditional" or "cultural" and "dirty food" emerged in the study and these were included in the model.

### Description of the research setting

This study was conducted in Uasin Gishu County in Kenya. Uasin Gishu County is one of the 47 counties of Kenya and is geographically located in the western part of the country. It has its headquarters in Eldoret town. The county is divided into six sub-counties and it covers a total area of 3,345.2 km2 with a total estimated population of 1,023,656, comprising 50% male and 50% female [32]. The predominant settlement pattern is rural (64.1%). There are 171 health facilities in the county, of which 90 are public [32]. Most of the facilities are concentrated within Eldoret Municipality. The climatic conditions and soil type in this region are generally favourable for a wide range of livestock and crop production.

The Kalenjin are the predominant ethnic population in Uasin Gishu County. This ethnic group is composed of smaller subtribes: the Kipsigis, Nandi, Tugen, Keiyo, Marakwet, Pokot also called the Suk, Sabaot and the Terik. The ethnic group has its own mother tongue. However the sub-tribes speak their own dialect. The Nandi have the highest settlement in the county, followed by the Keiyo [13]. The Kalenjin had been semi-nomadic pastoralists of long standing. They had been raising cattle, sheep and goats and cultivating sorghum and pearl millet since at least the last millennium B.C. The Kalenjin have a common staple diet: *Kimyet (ugali)* (a paste of cooked maize or millet flour sometimes mixed with sorghum flour), native vegetables and *mursik* (sour milk mixed with charcoal dust, sometimes mixed with cow’s blood), supplemented with roast meat (usually beef or goat). Fish was also part of the traditional diet though largely limited to residents bordering the lake region community. Childbirth among the Kalenjin was exclusively women’s concerns from which men were excluded. After delivery, women were considered unclean as a result they were secluded for a certain period of time during which, their movement was restricted to a given point within the household and were fed on special diet until the cleansing rite was performed.

#### Data collection

Semi-structured and key informant interviews were used for data collection in 23 rural health facilities selected from the six sub-counties of Uasin Gishu County between April and August 2015. Uasin Gishu County was purposively selected because this study is part of a broader project in the County on maternal and child nutritional health. The main factor considered in selecting the health facilities is that they must be in the rural area and have their population mainly composed of the Kalenjin clients, and be spatially distributed from each other to diversify responses.

#### Interviews with pregnant and post-natal women

Pregnant and post-natal Kalenjin women who came for routine antenatal and child welfare check-ups in the Maternal and Child Health (MCH) section in the rural health facilities were included in the study, based on availability and willingness to participate [33]. A combination of closed and open questions were used to investigate women’s subjective experience of pregnancy and nutrition [34]. The women were individually interviewed face to face in a private room at the health facility to give the opportunity to observe their facial expressions and body language, particularly important for correct interpretation of the answers [35]. Each woman was interviewed once by the first author and two female research assistants. The research assistants were well trained to ensure consistency and to ensure inter-researcher reliability. The interview guide was originally written in English but was translated into Swahili and into the local Kalenjin language where necessary. The local language interviews were conducted by the research assistants because they could speak and understand the language. The researchers obtained written informed consent before commencing with data collection. Respondents were asked about the following main issues: food that is restricted or recommended during pregnancy, and the underlying reasons, opinions on the beliefs and who gave the advice. Respondents’ social demographic characteristics were also captured. Each interview lasted for 25–90 min. None of the respondents pulled out of the interview before completion.

The data collection exercise was continued until saturation was reached [36] at a sample size of 154 responses. Out of the 154 respondents, only 42 gave consent to be audio taped; these 42 respondents provide the direct quotations in the results section. Most respondents feared to be audio-recorded, probably because many audio recorded witnesses to the 2007 post-election violence died mysteriously. As a result, for the other 112 respondents, extensive note taking was preferred to create a more relaxed interview setting and encourage full disclosure [36]. Demographic characteristics of these respondents are presented in Table 1 below.

**Table 1:** Demographic Characteristics of Respondents

Characteristic	Categories	Percentage (*N* = 154)
Maternal status	Antenatal mothers	40
	Post-natal mothers	60
Gravidity	First pregnancy/child	28
	More than one pregnancy	72
Age (years)	≤19	9
	20–24	29
	25–29	32
	30–34	18
	≥35	12
Marital status	Never married	18
	Currently married	77
	Separated	3
	Widowed	2
Educational level	Primary education	54
	Secondary education	34
	Post-secondary education	12
Occupation	Farming	40
	Business	21
	Housewife	18
	Formal employment	9
	Student/pupil	8
	Others	4
Ethnic Affiliation	Nandi	71
	Keiyo	18
	Marakwet	5
	Kipsigis	3
	Tugen	1
	Terik	1
	Pokot	1

Out of the 154 respondents, 62 were antenatal clients (ANC) and 82 post-natal clients (PNC) who had children younger than one year old. Various sub-ethnic groups of the Kalenjin were represented: the Nandi were the majority (71%) followed by the Keiyo (18%), the Marakwet (5%), and the Kipsigis (3%). The Nandi have the highest population in Uasin Gishu County followed by the Keiyo and the Marakwet [37]. There were two Tugen respondents, while Terik and Pokot were represented by one respondent each. The age of the respondents ranged from 18 to 42 years, with the majority of the respondents (61%) ranging between 20 and 29 years old. More than half (58%) of the respondents work in the domestic informal sector as either subsistence farmers or housewives. Most of them (88%) had not received more than secondary education. Most women were married (71%), while 18% were unmarried. Of the women who were unmarried, 50% were either pupils or students. 28% of the respondents were gravida 1.

#### Key informant interviews

In-depth key informant interviews were conducted with Traditional Birth Attendants (TBAs) who were also traditional pregnancy herbalists (*n* = 6), community health workers (*n* = 2), and the nursing officer in charge of MCH (*n* = 1). The nursing officer and the community health workers were selected from one of the largest facilities because they are likely to encounter a wide range of pregnancy nutritional experiences and challenges given their large catchment area. The TBAs were identified by snowball and convenient sampling through respondents who gave birth at home and who took herbal remedies during pregnancy. The TBAs were interviewed at their practise, either in their homes or market centres. Information gathered from key informants was employed to explore meanings and enrich the responses obtained from respondent interviewees. Key informants were interviewed about the following main issues: perceived restricted and recommended foods; in-depth information on underlying reasons for these recommendations; and their food advice to pregnant women. Each interview took 85–100 min. The key informants, particularly the TBAs, accompanied the researcher to the gardens to identify the food crops that were mentioned in the study, giving them their local names. The identified crops and animals were then photographed and collected for proper scientific identification and classification in consultation with the plant taxonomists at the University of Nairobi herbarium and the National Museums of Kenya

#### Data processing and analysis

Forty-two interviewees and most key informants (except the MCH nursing officers and community health workers) consented to being tape-recorded. The Swahili tapes were transcribed in English by the first author while the Kalenjin tapes were transcribed by a Kalenjin speaking transcriber. For the other respondents, extensive note taking took place. The transcripts and notes were coded and categorised in themes using the software for handling qualitative data MAXQDA (version 12.1.3) with each participant identified by a pseudonym. The pseudonyms are used in the narratives presented in the results section. The codes were then analysed for patterns, pre-set themes, emerging themes and categories including food that is recommended during pregnancy, food that is restricted during pregnancy and the underlying reasons, opinions on the beliefs and who gave the advice. The SPSS program (version 23) was used to establish the frequencies of descriptive statistics.

#### Ethical considerations

The research study was commenced after being approved by the National Commission for Science, Technology and Innovation (NACOSTI), Kenya. At the county level, research clearance was issued by the Uasin Gishu County Commissioner of Health, the County Administration Commissioner and the County Commissioner of Education. Request to access individual health facilities was granted by officers in-charge of the facility prior to the actual data collection day. The study was explained to each respondent and those consenting to participate were then requested to sign a written consent form. The respondents could withdraw from participation or answering further questions whenever they wished. The respondents’ identity was kept anonymous and their responses confidential.

## Results

Findings reveal that food beliefs are widely known and practised by pregnant Kalenjin women. Respondents mentioned various reasons for certain foodstuffs being restricted or encouraged during pregnancy as indicated in Tables 2 and 3. Food beliefs were mainly acquired from their *gogo* (their grandmother or any age-equivalent woman in the neighbourhood). They also learned food beliefs from their mothers, mothers-in-law, and other older female relatives, friends and neighbours. Only those with certain health problems (low haemoglobin status, low weight increase, hypertensive, diabetic or HIV positive) learned from health practitioners. Below, we present the results on pregnancy food restrictions, followed by food recommendations.

**Table 2:** Food restricted during pregnancy

Food type	Reason	Percentage (*N* = 154)
Animal organs^a^	Preserved delicacy for men and burren or menopouse women, tongue makes baby and women talk much	96
Meat^b^	Makes the baby big and brings misfortune to mother or baby	87
Eggs	Makes the baby big, causes high blood pressure and colic pain in the baby	68
Oily food^c^	Causes colic pain to the baby and makes the baby’s saliva ooze	25
Avocado	Makes the baby big	20
Alcohol and cigarettes	Results in a retarded, underweight and stunted child	19
Soil/soft stones^d^	Causes colic pain to baby and finishes baby’s blood	11
Sugary food^e^	Causes colic pain to baby, makes baby’s saliva drool	11
*Moboriet* ^f^	Makes the baby big, makes mother defecate a lot during birth, makes foetus docile	10
Fresh milk	Makes the baby grow big	7
Cabbages and kale	Are not nutritious, give the mother heartburn and nausea, use a lot of chemicals to grow	6
Salt	Makes the baby’s skin crack and peel, increases blood flow/pressure of the mother	5
Sheep’s head	Makes the baby have a blocked nose hence breaths with snoring sound	5
Cold water	Weakens the mother’s back ache after birth and prolongs labour pain	5
Plantain	Makes the baby big	4
Sheep meat	Causes allergies such as rashes to the baby’s skin	3
Vegetables grown on a burned soil	Cause burn-like rashes to the baby’s skin	3
Beans	Give the mother heartburn and nausea	3
Fermented milk	Gives the mother heartburn	3
Fermented porridge	Gives the mother heartburn	3
Rice and Irish potatoes	Supply less energy	3

^a^Tongue, heart, udder and reproductive organs (commonly obtained from either goat, sheep, cow or chicken)

^b^The common ones are obtained from sheep, goat, cow or chicken

^c^Using excess frying oil on food and food that require a lot of oil in preparation and cooking for example shallow and deep fried food

^d^This is pica, the craving and purposeful consumption of substances not commonly identified as food, is often reported among pregnant women

^e^Sweet food such as honey, soft drinks, sweet bananas, sugarcane, chocolates, sweets, tea or porridge with sugar

^f^Cold *ugali* (a paste of cooked maize flour, millet flour or sorghum flour, sometimes mixed with cassava flour), usually leftovers from the previous night and is normally taken in the morning as breakfast with other accompaniments

**Table 3:** Food recommended during pregnancy

Food type	Reason	Percentage (*N* = 154)
Traditional vegetables (Table 4)	Increase blood of the mother, gives strength to the mother and reduces heartburn	89
Milk	Gives strength to the mother, builds body and adds blood to the mother	63
Fruits^a^	Increase blood to the mother and protect the baby	35
Traditional herbs	Give strength to the mother, protect the baby, immunity booster to mother and foetus, reduce heartburn, ‘evil eyes’ and misfortunes and gastro-intestinal discomfort, increases uterine contractions during labour	34
*Ugali* ^b^	Gives strength to the mother	32
Porridge	Gives strength to the mother and increases the mother’s blood	32
Liver^c^	Increases blood of the mother	24
Water	Gives strength to the mother	15
Animal blood^d^	Increases blood of the mother	13
Beans	Gives strength to the mother and increases blood of the mother	12
Fish	Builds body and increases blood of the mother	11
Red soil and soft stones^e^	Increases blood to mother	7
Meat^f^	Adds blood to, and builds body of, the mother	7
Cooked bananas	Gives strength to the mother	5

^a^Oranges (Citrus sinensis), tangerine (Citrus reticulate), tamarillo (Solanum betaceum), passion (Passiflora edulis), pineapples (Ananas comosus), pawpaw (Carica papaya), Tamarillo

^b^Locally known as Kimyet a paste of cooked maize flour, millet flour or sorghum flour, sometimes mixed with cassava flour

^c^Commonly obtained from either sheep, goat, cow or chicken

^d^Obtained from either sheep, goat or cow

^e^Also called pica, the craving and purposeful consumption of substances not commonly identified as food

^f^Commonly obtained from goat, sheep, cow, or chicken

**Table 4:** Traditional vegetables

No.	Local name	English name	Botanical name
1	Mborochet	Pig weed	*Amaranthus dubuis*
2	chepkerta	Pig weed	*Amaranthus gracecizane*
3	Sochojek	African nightshade	*Solanum nigrum*
4	Sagek	Wild spiderflower	*Cleome gynandra*
5	Nderemiat	Vine spinach	*Basella alba*
6	Seveve	pumpkin leaves	*Cucurbita moschata*
7	Mrenda	jute mallow	*Corchorus olitorius*
8	Mito/miroo	Slenderleaf	*Crotalaria ochroleuca*
9	Kundek	Cowpea leaves	*Vigna unguiculata*

### Food restrictions during pregnancy

Several food avoidances were identified during the study and the underlying reasons for these practices are identified in Table 2 below. The foods to be avoided by pregnant women were organ meat (with exception of liver), mentioned by 96% of the respondents, meat (87%) and eggs (68%), oily food (25%), avocados (*Persea americana)* (20%), alcohol and cigarettes (19%), sugary food (11%), soil and soft stones (11%) and *moboriet* (left over *ugali* from the previous night commonly eaten as breakfast with other accompaniments) (10%). Other mentioned food restrictions were below 7%. Each of these (when above 10%) is explained below in order of frequency.

### Animal organs

Almost all respondents (96%) indicated that pregnant women are not allowed to eat animal organs, such as the tongue, the heart, the male reproductive organs and the udder (liver excluded). These organs are commonly obtained from a cow, goat, sheep or chicken. They are preserved for men and women are not allowed to eat them except barren women or those in menopause. All respondents reported that they have not eaten them since childhood. Some reasons given for this were as follows.

*“I think you can become barren if you eat the reproductive organs.”* (150602_001-80)

*“My father told me that my baby will talk much if I eat the tongue.”* (150514_005-88)

Furthermore, a number of respondents felt that such restrictions were put in place by male members of the Kalenjin ethnic group to protect animal organs from being consumed by women for their own selfish gain. A key informant reported that if a woman eats any of these organs, even in the absence of men, she will be considered disrespectful because she is showing dominance (proving to be the ruler of the homestead) which is very wrong. In addition, some respondents reported that if a woman eats animal tongue, she will talk more than men when women are supposed to listen while men talk, both at the homestead or in a public gathering. Therefore, these animal organs were either to be thrown away or given to a man in the neighbourhood if men (husband or senior sons) are not present. Another key informant reported that a Kalenjin man’s life traditionally evolved in the forest (a dangerous zone with wild animals) either hunting, fighting, herding animals or in search of stolen animals. Thus, eating animal heart made them develop a strong and bold heart to face the tough challenges associated with forest life.

### Meat

Women were advised to be cautious about eating any type of meat during pregnancy as reported by 87% of the respondents. The main reason for restricting meat was based on reasons related to the condition of the animal during life or upon death. Many respondents thought that the condition could in several ways be transferred to the pregnant mother and/or her unborn child. First, an animal that ever encountered pregnancy-related complications, such as miscarriage, stillbirth or death due to placenta retention, should not to be eaten. When a pregnant woman eats such meat, it was believed the animal will transfer “bad blood” to the mother and she will encounter similar complications during pregnancy and childbirth:

*“If a cow is pregnant and a calf dies in the uterus, a woman should not eat it. Also, if a cow was giving birth and the placenta refused to come out a woman should not dare eat it. If you eat it, you will be like that cow”.* (150504_001)

This belief is strongly observed by the Kalenjin community, especially the Marakwet and the Keiyo. All respondents who held this belief did not eat such meat during pregnancy. In addition, pregnant women are not allowed to eat meat of an animal which died without being slaughtered because of the possibility of passing on a dangerous disease to the unborn child or the mother.

Furthermore, women refrain from eating meat because it may have unknown negative associations, for example:

*“If a cow is born lame, when slaughtered you cannot eat it, we believe you may give birth to a lame baby. Even a stolen animal is not allowed, you will give birth to a thief.”* (150621_001-53)

Among the Kalenjin community, it is a taboo for pregnant women to eat the meat of an animal which died by strangling because it is believed that the spirits of this animal will follow the baby and kill it by coiling the umbilical cord around the baby’s neck.

A number of respondents also noted that an animal that was struck by lightning could only be eaten by men because it would kill women:

*“Meat of an animal killed by lightening is not even allowed to be brought into the house; it is slaughtered and eaten by men at the striking spot. The leftovers including bones should be burned beyond recognition. …It can cause death to a pregnant woman if she eats it”.* (150610_003-77)

Therefore, if a pregnant woman wants to eat meat she needs to know the history of the animal:

*“If you are pregnant and you are not sure of a certain meat, don’t attempt to eat it, you ask first. But this is something everybody knows, even the owner of the meat will not let you eat it or buy it if he notices that you are pregnant. He will stop you.”* (150602_001)

In case a woman eats such “evil” meat by mistake she has to undergo certain cleansing rituals.

*“If you accidentally eat an animal that was unable to deliver and died, they must do for you cleansing rituals. This will involve people from the husband side and the wife side. After the rituals, nothing will happen to you”.* (130709_006-129)

A key informant explained that some of the herbs consumed during pregnancy neutralise the effect of this “evil meat” if eaten unknowingly by the mother.

In some cases respondents referred to specific kind of animals that should not be eaten. Some respondents reported that a pregnant woman should avoid beef, because cattle receive routine treatment, such as injections and immunisations that could be a health hazard to the foetus.

*“They restrict meat from cattle too, but sheep, goats are allowed… personally I did not eat it. It is not allowed, because cattle are given a lot of drugs.”* (130709_002-126)

Eating mutton during pregnancy, as reported by 3% of the respondents, will cause health problems in the new-born baby:

*”I did not eat mutton, even ‘gogo’ told me not to. You know if you eat mutton, when you give birth, the meat brings rashes on your baby’s skin, like those of an allergy.”* (130707_005-106)

*“Meat of sheep’s head is not allowed to be eaten by a pregnant woman, the child will have nasal block. Have you ever heard a child snoring strongly even when awake? They say it’s because you ate sheep’s head during pregnancy.”* (150510_003-1)

Several respondents noted that meat should not be eaten “in excess” by a pregnant woman – at most once a week because it has proteins that make the baby grow big, causing problems during birth.

### Eggs

Eggs are believed to be either “too strong” or “too heavy” or to have “too much protein” and are hence not good for a pregnant woman. This view was shared by 68% of the respondents. In this study, it was believed that strong foodstuffs such as eggs are believed to make the foetus grow excessively big and this will cause problems to the mother during birth. They further had different views about eating eggs as stated below:

*“We are told if you eat eggs, they will bring problems on the day of birth, the baby will defeat you, and you will undergo an operation* [caesarean section].*”* (150510_002-25)

*“They say that if a woman is pregnant and eats eggs, there are high chances of developing high blood pressure.”* (130707_004-105)

*“You know if you eat eggs, the baby will develop stomach pain, and she/he will be crying a lot after birth.”* (150510_003-1)

A pregnant woman should preferably avoid eggs completely during the entire period of pregnancy. If it is essential that a woman eats eggs (for example due to hospital recommendations or pregnancy “urges”) she should only eat a few eggs or mix them with other food such as *chapati* (Flat round shallow fried substance made from wheat flour, salt, sugar and oil) or vegetables to neutralise the effect:

*“I find it a wise idea not to eat eggs when pregnant. You should eat may be once, … say after a month or two months if you have that strong urge, otherwise, you are supposed to control yourself”.* (130707_004-105)

*“I ate twice for the entire pregnancy period but I did not eat them plainly. I mixed them in dough for making ‘chapati’ and in ‘sukuma wiki’ [Brassica Carinata].”* (150610_001-75)

Some respondents mentioned that the effect of eggs is more severe at certain stages of pregnancy although these stages differed:

*“We are restricted eggs from six months onwards.”* (130707_008-107)

*“A woman cannot eat eggs from the time of conception but after finishing 6-7 months it is allowed.”* (130709_006-129

### Oily food

Some of the respondents (25%) reported that “too much oil” is not recommended for a pregnant woman. It is considered “too much oil” when food is prepared using a lot of oil, especially deep frying. The reported foods of this kind include *chapati*, *mandazi,* 1 *kangumu,* 2 eggs, French fries and fatty meat. The main reason for avoiding fatty food is that it causes colic pain to the baby. It can also make the baby fat (making the birth difficult), and cause high blood pressure and malaria in the mother:

*“The food that has a lot of oil for example eggs, ‘kangumu,’ ‘mandazi’, or fatty meat, you should not eat much. … I don’t know what it has that if you eat, the day of delivery it will bring problems, you will undergo an operation.”* (150510_002)

*“You should use very little oil in frying food, not excess because a pregnant woman’s blood already flows very fast. If you add oil on top, the heart will beat excessively.”* (130707_002-104)

### Avocado (Persea Americana)

Avocado is also restricted during pregnancy as reported by 20% of the respondents. Avocado is believed to have similar effects as eggs. It causes the baby to grow “fat”, causing delivery problems. It should therefore be avoided completely or eaten once in a while (one in a week).

*“Avocado can hurt you … This is something I was told by my mum and I believed it. … If you eat it, you are causing problems to yourself, you might tear during birth or you will not be able to push out the baby.”* (150617_002-9)

“*They have said I should avoid it [avocado], though I used to it eat because I didn’t know it was wrong until I was stopped. … it is a good idea, because if you eat, the baby will grow …, and if the baby grows big, definitely you will go to theatre.”* (150621_001-53)

“*Avocado you only eat once in a week. I personally did not eat it completely. I find it an okay advice, because if you eat, the baby will be too heavy and you will not be able to deliver.”* (130709-002-126)

### Alcohol and cigarettes

According to 19% of the respondents alcohol and cigarettes are restricted. They are believed to cause low birth weight and stunted children because they “suck the baby’s blood”. They also affect the baby’s brain, causing retardation in children.

### Sugary food

Other respondents (11%) reported that “a lot of sugar” is not good when a woman is pregnant. Sugar in this case refers to sugar plus any foodstuffs known to be “sweet”, including (in order of prevalence) sugarcane, honey, sweet/ripe bananas, sweets, chocolates, and sugared beverages and drinks such as tea, soda and processed juice. Women held a belief that the baby will salivate excessively if the mother had ingested sugary foods during pregnancy. Sugary foods are also believed to give the baby colic pain and may give mother and baby malaria:

*“If you eat sugarcane, your child will have this thing… we call it ‘meninek’ I mean will be oozing a lot of saliva.”* (150615_002- 98)

*“We call it ‘surunda’ (*colic pain) *this stomach that disturbs newborns. Ripe bananas are not good.”* (130708_001-118)

### Soil and soft stones

During pregnancy, some women crave and purposefully consume non-food items such as soil, chalk, laundry starch, ice, or cigarettes which they claim has a good taste or smell. This habit is commonly known as *pica* (a Latin word for magpie, the bird notorious for eating almost anything) and the patterns of ingestion are referred to as “*Phagia”* [38]. In this study, 11% of the respondents reported *pica*. Often reference was made to the practice of eating clay soil, commonly obtained from termite mounds or dry mud walls of houses (geophagia) and soft stones (lithophagia). These stones are mostly light-yellow in colour and are sold in open-air markets, supermarkets or can be picked along the way. Ingestion of soil and soft stones is a common behaviour among women in Kenya, especially during pregnancy [38–41]. However, in this study, many respondents reported that soil and stones should be avoided during pregnancy because they would give newborn babies colic, cause anaemia in the mother, and the mother will give birth to a baby who will be eating soil.

*“I did not eat soil so my baby had this normal stomach problem, but if you add the soil on top the problem becomes excess.”* (150617_002-9)

*”I was having four pints of blood (Hb4) when I was pregnant of my other daughter … And that is why I was telling you that soil finishes blood because I used to eat a lot of it even before I conceived that pregnancy.”* (130707_005-106)

A key informant reported that if burnt soil (obtained from wall of burned mud house and clay cooking stones) is consumed by a pregnant woman, the child will develop burn-like skin rashes which cannot be treated at the hospital and which can only be cured using traditional herbs mixed with a powder of burnt soil.

### Moboriet

Another pregnancy food restriction mentioned by 10% of the respondents was *moboriet. Moboriet* is *ugali* left over from the evening meal. Among the Kalenjin, *moboriet* is commonly eaten with hot tea or fermented milk as breakfast, especially by women in most rural homesteads. Respondents consider that consumption of *moboriet* by a pregnant woman will make the foetus oversleep, docile in the womb, fat and difficult to deliver. It was also mentioned that *moboriet* will cause a pregnant woman to defecate during birth, troubling the midwife:

*“You should not eat* moboriet *when you are pregnant especially in the morning. A woman poops all the time during birth before the baby comes out. They [the midwives] will be wiping it off every time that is why they do not let us eat it.”* (150609_002-89)

### Other pregnancy food restrictions among the Kalenjin

Respondents reported many other food restrictions, although these were less frequently mentioned (<7% of the respondents). For example, fresh milk, especially when hot, was believed to make the baby grow big and difficult to deliver. Instead, *mursik* (fermented milk) was recommended. However, others reported that *mursik* gives pregnant woman heartburn hence fresh milk is preferred. Beans, fermented porridge and kale *(Brassica Carinata)* are also associated with heartburn during pregnancy. Three women believed that taking salt or soda ash during pregnancy will cause the baby to have rough, dry and cracking skin which will eventually start to peel off like that of a snake. Other foodstuffs such as wild cabbages (*Brassica oleracea)* and kale *(Brassica Carinata)* are believed to be nutritionally less valuable foods while rice (*Oryza sativa)*, Irish potatoes *(Solanum tuberosum)* and plantain *(Musa × paradisiaca)* are believed to be ‘light’ food, supplying less energy, and should not be frequently consumed by pregnant women.

Eating vegetables grown on burned ashes or burned soil is believed to cause dangerous burn-like rashes all over the skin baby’s skin, similar to those mentioned earlier that are caused by eating burned soil. Drinking cold water during pregnancy and during labour is restricted because it is believed to weaken the mother’s back and prolong the labour pains.

#### Food types recommended during pregnancy

The types of food items recommended for consumption by pregnant women, and why they are considered important, are presented in Table 3 below.

Four dominant nutritional beliefs attributed to the recommended foodstuffs emerged. These types of food were believed to either “increase blood”, “increase mother’s strength”, “build the body of mother” or protect the mother and child from disease. The commonly cited food recommended for pregnancy include traditional vegetables (89%), milk (63%), fruits (35%), traditional herbs (34%), *ugali* (32%), porridge (32%), liver (24%), meat (18%), water (15%), animal blood (13%), and beans (12%). Some of these are discussed in more details below.

##### Traditional vegetables

The consumption of traditional or indigenous Kenyan green vegetables during pregnancy is one common cultural food belief and practice that was reported by almost all respondents (89%). The consumption of traditional vegetables is not only highly recommended by the *gogos* but it is also advised by health workers as a nutritional therapy for pregnant women with lower Hb levels. Traditional vegetables identified by respondents include pigweed (*Amaranthus dubuis* and *Amaranthus gracecizane)*, African black nightshade (*Solanum nigrum*), wild spider flower (*Cleome gynandra),* vine spinach (*Basella alba*), pumpkin leaves (*Cucurbita moschata),* slender leaf (*Crotalaria ochroleuca)* and cowpea leaves (*Vigna unguiculata*). Spinach is the only exotic green vegetable recommended for increasing the volume of blood. All the recommended vegetables are deep green and leafy. They are grown in gardens and farms, although some also grow as weeds along footpaths and in uncultivated fields. Mostly they do not require maintenance use of pesticides and artificial fertilisers. As a result, these vegetables are widely available within Uasin Gishu County. The vegetables are prepared as a sauce with cream obtained from cow’s milk, salt, and tomatoes. No oil is added but a few mentioned enriching it by adding animal blood when cooking.

These vegetables were generally considered to “increase the volume of blood” in a woman’s body and to “build body” which gives women strength during labour. Unlike other exotic vegetables which grow in the study area, such as wild cabbage (*Brassica oleracea)* and kale *(Brassica Carinata),* traditional vegetables are not believed to cause heartburn and they are considered to digest easily, relieving constipation. For these reasons, traditional vegetables are preferred by all members of the community. Various respondents reported the importance of eating traditional vegetables:

*“This* nderemiat *[Basella alba] and* miroo *[Crotalaria ochroleuca) are very helpful, when you eat them you do not feel heartburn.”* (150602_001)

*“When you are pregnant and your blood is low, you must eat these vegetables so that your blood can increase.”* (150617_006-13)

##### Milk and blood

Milk, according to 63% of the respondents, is believed to “increase the volume of blood”, “give strength” and “build the body” of the mother. To boost its nutritional value, milk is mixed with raw blood extracted from a healthy bull or collected from the slaughterhouse. Milk mixed with blood is believed to raise blood volume very rapidly. As a result, it is highly recommended for women who were informed at the clinic that their Hb is low:

*“When you are pregnant and you are told your blood is less and should be increased, you need to go to the butchery, collect blood of the cow, sheep or goat depending on what animal they slaughtered, ferment it with fresh milk, stock and you be drinking it daily. It helps your blood volume to increase fast.”* (130707_003-104)

Animal blood can also be consumed without milk. It is normally cooked until it hardens and served as a meal. The consumption of blood is a prominent Kalenjin custom of the past as explained by a key informant. However, the consumption of blood is being discouraged by the public health officers on the grounds that animals receive injections in various treatment regimes that might not be good for human health.

##### Fruits

Some 35% of respondents reported that eating fruit, with the exception of avocado and ripe bananas, was recommended because fruit is believed to increase the mother’s blood volume. The commonly reported fruit known to increase blood were the tamarillo *(Solanum betaceum)* and oranges (*Citrus sinensis*). Other fruits mentioned in the study include: tangerine (*Citrus reticulate*), passion fruit *(Passiflora edulis),* pineapple *(Ananas comosus)* and pawpaw *(Carica papaya*)*.*

Fruits are also believed to improve mother’s appetite, help in digestion, and improve the skin of the baby after birth.

##### Ugali

Thirty two percent of the respondents reported that Ugali is recommended for pregnancy. *Ugali* is the local staple food among Kenyan communities including the Kalenjin. It is most often made from maize flour, but also from millet or sorghum flour, or a mix of cereals, and is cooked with water until a dough-like substance is attained. Maize is affordable and widely available in Uasin Gishu County and is grown for subsistence as well as commercially. *Ugali* is consumed with vegetables, fermented milk, or meat stew. *Ugali* made from a mixture of sorghum and finger millet flour is highly recommended during pregnancy. This type of *ugali* is believed to be nutritious and “stronger” than the *ugali* made from maize flour. The majority of the respondents believe that *ugali* gives strength to the mother:

*“Ugali is good. It gives you energy the day of pushing the baby out.”* (150602_001)

##### Traditional herbs

Consumption of traditional herbal medicine is also highly recommended for pregnant women as reported by 34% of the respondents. The traditional herbal medicine comprises 10 different mixed plants. A key informant (herbalist who supplies herbs to pregnant women) explained that the plant extracts (roots, leaves, twigs or bark) are either boiled until they reduce to a small concentrate (500 ml) locally known as *sagetiek* (in Nandi language) or are burned to form an ash-like powder (known as *bosarok*). It is recommended that a woman consumes a quarter of a glass of *sagetiek* three times a day for two weeks, break for a month and then resumes with a similar dose. It is also consumed two days before the due date and when the mother is overdue. *Sagetiek* is either taken alone, or in some cases combined with honey or soup if extracted from bitter plants. The soup is made from the head and hooves of an animal, preferably a goat or cow. On the other hand, *bosarok* is licked daily for a prescribed period because it is believed to protect pregnant women from an evil eye and witchcraft that might affect the unborn child. Respondents consider that *bosarok* is very useful, especially when the pregnant women expect to meet a lot of people who might put a curse on the baby. In addition, *bosarok* is also considered to relieve heartburn and reduce nausea.

Women can purchase herbal remedies from a known herbalist in the village. The herbs are believed to be useful and it is generally considered that they should be consumed by a pregnant woman whether sick or not. *Sagetiek,* as explained by key informants, is believed to protect pregnant woman from getting sick and from transferring sickness to the foetus. It is also believed to make the woman strong and increase blood volume, facilitating easy birth by accelerating labour and cervical dilation. It is also believed to build the body and health of the foetus, change the sex of the foetus from girl to boy, neutralise the effects associated with eating “evil meat”, and regulate the size of the baby. The herbs are also believed to clean the foetus’s skin and stomach, give the baby smooth, rash-free skin, reduce a baby’s colic pain and decrease its vulnerability to communicable diseases when born. Thus, *sagetiek* is widely recommended for pregnant women*.*

##### Porridge

Recommendation of porridge during pregnancy was reported by 32% of the respondents. Porridge, when prepared from the flour of finger millet mixed with sorghum, is believed to increase the volume of blood. In addition to boosting blood volume, porridge is believed to give pregnant woman strength hence it is particularly recommended for consumption during breakfast and in-between the meals.

##### Liver

Twenty four percent of the respondents reported that liver is highly recommended for pregnancy because it increases the volume of blood. Whenever an animal is slaughtered in the village, liver is specifically reserved for any pregnant woman or children within that homestead. Liver is also advised by health workers to pregnant women with lower Hb levels.

##### Other food recommendations

Other recommended food stuffs which were reported by less than 20% of the respondents, including meat, soup of red beans, fish, red soil and red stones, are believed to increase pregnant woman’s blood volume. Cooked bananas and water during pregnancy are believed to increase the strength of the mother. In addition, when an animal is slaughtered (mostly goat, sheep, cow or chicken), its liver is preserved for any pregnant woman in the homestead. It is believed to increase the volume of blood hence recommended for pregnant women.

## Discussion

The results demonstrate that food beliefs and practices are still commonly subscribed to and practised by a large majority of the contemporary Kalenjin pregnant women of Uasin Gishu County. These findings are comparable with those of Ekwochi et al. [23] and Zepro [25] who found that more than half of the women in south-east Nigeria and Ethiopia, respectively, had traditional food beliefs.

Protection of human health emerged as the dominant reason for practising food beliefs in this study. Protection of health conditions were also stated in the literature as reasons for the prevalence of food taboos in Ethiopia and Gambia [24, 25]. This is unlike other regions of Zambia where fear of foetal malformation and abnormality was established to be the major reason for pregnancy food beliefs [42]. Varied other reasons for subscribing to food beliefs during pregnancy emerged from the study as described below. This supports literature that indicates there is no single reason for the belief and adherence to food taboos [43].

### Health and illness as determinants of food selection and restriction

The Kalenjin women adhere to food beliefs as a way of avoiding any calamity that might interfere with pregnancy and child birth. Child bearing among the Kalenjin is very important because it is the dominant way a woman acquires bride price, gains security, acceptance and identity in the family and the community [44]. Children constitute the most important visible sign of success and achievement in the society to both husband and wife [44]. Pregnancy food precautions as a concern for a healthy pregnancy and birth outcome was also established in Nigeria, Ethiopia and the Gambia [23–25]. Four principle lines of reasoning emerged from the responses that are believed to facilitate easy labour and birth, as indicated below:

First, the foetus should be kept small. It is considered that large foetuses are difficult to deliver, resulting in episiotomy, prolonged and obstructed labour and possibly caesarean section (CS), increase chances of death of the mother or child. Eggs, oily food, meat, fresh milk, *Moboriet* and cooked potatoes during pregnancy are believed to make the foetus grow excessively big, hence these foods are restricted. Prescription of herbs was also established as a remedy for regulating the size of the baby and to accelerate contractions during labour.

This study was carried out in a rural area where the largest accessible health institution is a health centre which only conducts normal deliveries, primary healthcare and basic first aid for obstetric complications. It is headed by a Clinical Officer who is not qualified to conduct surgery in case of an emergency [45]. It has also been established that the referral system in case of an emergency is very poor, exacerbated by poor rural road access [45, 46]. Hence, respondents’ fear of a complicated delivery is justified. Restricting the foetus from growing large as a way of facilitating easy birth was also established in Ghana, Ethiopia and South-East Nigeria [21–23, 25]. Consumption of cow’s blood, sour milk and much water, as well as vomiting after a heavy meal, is also practised by the Maasai women in Kenya, also aiming to keep the baby small at birth to facilitate delivery [29].

According to the respondents, the occurrence of easy labour and birth is also determined by the strength of the woman. They consider that a weak woman cannot push out the baby easily and that she can even faint or die in the process. Such a woman can only be helped by CS which is, as mentioned above, believed to be a risky endeavour. For a woman to have enough energy reserves during birth, she is expected to consume food believed to give strength during her pregnancy period. Such food includes *ugali* and porridge made from finger millet mixed with sorghum, and traditional vegetables, milk, traditional herbs and meat. Similarly, Towns [19] also established how Beninese and Ghanian women consumed medicinal plants to strengthen women during pregnancy and that delivery would be facilitated through consuming these plants. On the other hand, energy-giving starch-rich items were regarded as good for body among rural Nigerian mothers [47].

Another factor that is believed to facilitate easy labour and birth is to have sufficient blood. Fewer blood reserves make a woman weak. A woman with less blood is also believed to bleed excessively during labour, birth or after birth, which may necessitate a blood transfusion or cause death if she cannot access a health facility in time and get a willing blood donor. According to many respondents, the prominent way of ensuring enough blood is consuming plenty of traditional vegetables, liver, animal blood, fruits, milk, beans, red soil/stones and fish during the pregnancy period. On the other hand, a woman should avoid eating soil and soft stones (pica) because they are also believed to “suck” her blood (causing anaemia). Ingestion of *pica* involving soil and soft stones is a common behaviour among women in Kenya especially when pregnant [38–41], possibly representing a natural response to a nutritional depletion, such as iron deficiency [39, 48]. Although consuming clay soils has been found in the literature to be medicinal [49], it can lead to reductions in birth weight, haemoglobin, haematocrit, and red blood cell levels, thus causing maternal anaemia [50]. Iron and folic-acid deficiency anaemia during pregnancy is very common in Kenya [51] and is associated with maternal death [28] hence the fear of iron deficiency among these women is justified. A belief in the importance of iron was also established among pregnant women in Ghana where, likewise, traditional green vegetables were believed to provide blood or prevent anaemia [20].

Evil spirits or supreme powers are also believed to interfere with good health outcomes by causing misfortune and complications during pregnancy, labour or birth. Death of an animal is sometimes believed to be caused by evil spirits and these evil spirits are transferable to humans. Therefore, if a woman eats the meat of a dead animal killed by evil spirits, these spirits can cause a similar death or misfortune to the mother or child. The commonly established powerful spirits are those that cause animal death through pregnancy-related complications such as miscarriage, stillbirth, retained placenta, foetal malpresentation or that cause an animal to hang itself, stricken by thunder, sickness, or animal deformity. Fieldhouse [52] identified food restrictions related to magical thinking in which some plants and animals (e.g. dogs, cats, pigs) were perceived to have spirits and were therefore not consumed.

Foods culturally recognised to cause “illness” were established to influence food choices in this study. Some foods were restricted because they are believed to cause sickness to mother and child: including neonatal death, skin rashes, colic pain, and nasal block or breathing difficulties in the infant and nausea feelings, vomiting, miscarriage, preterm birth and maternal death to the mother. Foods believed to cause these illnesses to the child include foods that are oily, sugary and salty. Eating soil, stones, mutton, sheep’s head and vegetables grown on burned soil are also believed to cause these childhood illnesses. Notably, these illnesses which are caused by eating of restricted foods are not bio-medically treated. Instead, they are treated locally by use of various herbs. Studies have identified a comprehensive list of medicinal plants used by the Kalenjin herbalists to treat several ailments including skin rashes, colic pain, infertility and miscarriage [53, 54]. However, these studies tend to overlook the safety and efficacy of using these herbs and how herb consumption influences or prevents illness. A study by Towns [19] in Ghana established that most herbal plants have antibacterial, anti-inflammatory, antimicrobial and antimalarial activity. Use of herbal medicine to treat pregnancy-related health ailments is a common practice in rural and urban Kenya [55–57] as well as in Europe, Australia, Zambia, South Africa and Ghana [19, 58–60].

Some foods are not restricted by culture but are believed to cause discomfort, such as heartburns, nausea or vomiting, a condition the respondents referred to as “being rejected by the foetus”. Commonly cited foods in this category include fermented poridge, fermented milk, cabbages, kale, beans and *githeri* (maize mixed with beans). Consumption of traditional vegetables and fresh milk is believed to relieve heartburns, nausea and vomiting, and is encouraged. Food cravings or rejection due to physiological changes that resulted in overconsumption or underconsumption of nutritious or non-nutritious food has previously been established by Aikins [20] among pregnant women in Ghana.

### Gender and age as determinants of food selection

Some food stuffs were also judged to be more or less appropriate for certain classes of individuals in the society. Attributes of “male” versus “female” foods, their symbolism, and how they affect actual consumption of food were also established in the study. For instance, the Kalenjin men reserve some animal organs such as the tongue, heart, male reproductive organs and udder for themselves as delicacies; these foods are taboo for women and children. On the other hand, liver is reserved for any pregnant woman in the family. Subdivision of animal body parts/meat according to age and gender is a common cultural practice among the pastoralist communities in Kenya, including among the Kalenjin. Similarly, the Luhya community of Kenya restrict consumption of eggs in order to spare chickens because chicken meat is a delicacy reserved for men and guests [29]. Such a practice can result in a shortage of adequate supplies of essential nutrients, especially among vulnerable groups.

### Rituals as factors in food selection and avoidance

In this study, herbal medicine, milk, animal blood and traditional beer were commonly used in conducting cleansing rituals when pregnant women accidentally consume restricted food stuffs. Herbal medicine as explained by a herbalist in this case is consumed by the pregnant women whereas traditional beer, animal blood and milk were moth sprayed at the grave yard as a reconciliatory negotiation between the pregnant woman and the ancestors. as a result, these herbal trees are domesticated in the gardens because they are getting diminished in the forests due to deforestation. The role of taboos as a protection of ritual plant species was also established by Quiroz in Benin and Gabon [16].

### Food beliefs observed to enhance decency and aesthetics

Some food restrictions are based on decency and aesthetics. Consumption of *moboriet* is believed by some to make a woman defecate during birth. At the same time, sugary food is believed to make the baby messy by encouraging saliva over production hence drooling.

#### Food beliefs to enhance group identity and cohesion

The majority of the pregnancy food recommendations were indigenous to the Kalenjin diets generally and were termed as traditional foods (for example animal blood, milk mixed with blood, milk, green leafy vegetables, meat, fruits, beans, *ugali* and porridge made of finger millet and sorghum flour). The majority of the indigenous foods discussed cut across the sub-groups of the Kalenjin community. This signifies cultural identity, continuity, cohesion and a feeling of belonging and uniqueness in food consumption patterns in these Kalenjin sub-groups. It also probably indicates that pregnancy diet patterns have not changed much over time.

One of the limitations of this study is that the implications of the food beliefs during pregnancy on the nutritional status of Kalenjin women were not examined because it was not a research objective and because the study was subject to time and resource constraints. Future studies should use a combination of analysis of dietary intake such as food frequency questionnaire, food diary and 24-h dietary recall to determine the actual nutrient intake and nutritional status of the women.

## Conclusions

The results of this study demonstrate that food beliefs and practices are still commonly subscribed to and practised by a large majority of the contemporary rural Kalenjin pregnant women. Some food beliefs such as eating traditional vegetables and milk, as well as restricting the consumption of organ meat (with exemption of liver), meat and eggs, are subscribed to by more than 60% of the respondents. Consumption of fruits, traditional herbs, *ugali,* porridge and liver, and restriction of avocadoes and oily food was reported by more than 20% of the respondents. The reasons cited for adherence to these dietary precautions were largely based on protection of human health, mainly in realising a successful pregnancy and delivery. Sufficient maternal blood, energy and restricting foetal size and evil contamination were believed to result in a successful pregnancy outcome. Protection of foetal ill health, such colic, skin rashes and malformations was also established, though much less frequent than the health of the pregnant woman. Other reasons such as monopoly of a scarce resource (organ meat), decency and aesthetics also emerged. Based upon these conclusions, we recommend the following:

- Considering the high prevalence of food beliefs, it is advisable that when nutritious foods are restricted, nutritional interventions should rather search for alternative sources of nutrition which are available and considered to be appropriate for pregnancy.
- In this study, we also found ambiguity in the food beliefs. Some food such as meat, beans, cooked bananas, soil, and milk were reported by some respondents as restricted, while these were mentioned by others as being recommended. Intervention programs should thus design nutrition training tools tailored to these intra cultural variations in food beliefs.
- Some of the beliefs, such as consumption of traditional vegetables, milk, beans, liver, fruits, porridge and *ugali* as well as the restriction of eating soil and soft stones, meat of a dead animal, excess salt, excess sugar, excess oil, alcohol and cigarettes, are supported by science and are indeed beneficial for health, hence need to be encouraged. However, it should be clarified how much should be consumed. On the other hand, restriction of nutritious food such as eggs, meat, avocadoes, animal organs, vegetables planted on ashes, as well as recommendation of red soil, need to be sparingly discouraged.
- Traditional herbs are widely consumed during pregnancy not only in Uasin Gishu but also in other parts of Africa. There is the need to study the chemical constituents of these herbs to learn how safe and relevant they are for human consumption, especially during pregnancy and foetal development.
- The major reason for food beliefs and adherence to them is to protect health of the mother and child, and ensuring a successful pregnancy outcome. Intervention programmes that provide nutritional advice need to address these health concerns and the assumptions that underlie successful pregnancy and delivery, given the deep-rooted nature of the beliefs.

## Abbreviations

ANC: Antenatal clients
CS: Caesarean section
FAO: Food and agriculture organization
HB: Haemoglobin
HIV: Human immunodeficiency virus
IFAD: International fund for agricultural development
IUGR: Intra uterine growth restrictions
KNBS: Kenya national bureau of statistics
MCH: Maternal and child healthcare
NACOSTI: National commission for science, technology and innovation
PNC: Post-natal clients
SEKU: South Eastern Kenya university
TBAs: Traditional birth attendants
UNICEF: United nations international children’s emergency fund
VU: Vrije Universiteit Amsterdam
WFP: World food program

## Footnotes

1 Also known as *Swahili bun*, deep fried bread, similar to doughnuts, made from wheat flour mixed with salt, sugar and oil to taste.

2 A type of *mandazi* but very compact and hard with no airspaces inside, made from wheat flour mixed with salt, sugar and oil to test and is deep fried
